# The influence of social capital on employers’ use of occupational health services: a qualitative study

**DOI:** 10.1186/s12889-015-2416-8

**Published:** 2015-10-23

**Authors:** Christian Ståhl, Carl Åborg, Allan Toomingas, Marianne Parmsund, Katarina Kjellberg

**Affiliations:** Department of Medical and Health Sciences, National Centre for Work and Rehabilitation, Linköping University, Linköping, Sweden; Department of Psychology, Uppsala University, Uppsala, Sweden; Institute of Environmental Medicine, Unit of Occupational Medicine, Karolinska Institutet, Stockholm, Sweden; Department of Occupational and Environmental Health, Stockholm County Council, Stockholm, Sweden

**Keywords:** Occupational health, Sick leave, Return to work, Prevention, Organizational policy, Sweden

## Abstract

**Background:**

Occupational health services may have a strategic role in the prevention of sickness absence, as well as in rehabilitation and return to work after sick leave, because of their medical expertise in combination with a close connection to workplaces. The purpose of this study was to explore how employers and occupational health service providers describe their business relations and the use of occupational health services in rehabilitation in relation to the organization of such services. The study uses a theoretical framework based on social capital to analyse the findings.

**Methods:**

Interviews and focus groups with managers with Swedish public employers (*n* = 60), and interviews with occupational health services professionals (*n* = 25).

**Results:**

Employers emphasized trustful relationships, local workplace knowledge, long-term contracts and dialogue about services for good relationships with occupational health providers. Occupational health providers strove to be strategic partners to employers, promoting preventive work, which was more easily achieved in situations where the services were organized in-house. Employers with outsourced occupational health services expressed less trust in their providers than employers with internal occupational health provision.

**Conclusions:**

Social capital emerges as central to understanding the conditions for cooperation and collective action in the use of occupational health services, with reference to structural (e.g. contracts), relational (e.g. trust) as well as cognitive (e.g. shared vision) dimensions. The study suggests that attention to the quality of relationships is imperative for developing purposeful occupational health service delivery in rehabilitation and return to work.

## Background

Research on rehabilitation and return to work (RTW) for people with work disabilities has concluded that early workplace-oriented multidisciplinary interventions are advised, in cooperation with health care, employers, insurers and the person off work [1, 2]. Studies have shown that employers find reintegration of workers after disability challenging [3]. In this respect, occupational health (OH) services may have a strategic role in preventing sickness absence, as well as in rehabilitation and RTW after sick leave, because of their medical expertise in combination with a close connection to workplaces [4, 5].

However, in order to use OH services strategically in rehabilitation processes (or in preventing sick leave), the relationship between employers and OH service providers needs to be purposive, and the provision of services needs to be arranged in a way that facilitates such a relationship. Earlier studies have pointed out that OH involvement in rehabilitation is often lacking [6]. The organizational conditions for involving OH services in rehabilitation and RTW have this far received little research attention.

### OH services in Sweden

Access to OH services differs greatly between countries. In some countries, occupational health aimed at RTW barely exists, whereas in others it is obligatory by law for employers to have an OH affiliation (e.g. Finland). In the Netherlands, all sick-listed persons are required to visit occupational physicians for rehabilitation purposes [7, 8]. In the United States, OH is typically used either in connection with workers’ compensation laws to provide medical services for workers with occupational injuries or illnesses, or to provide general medical services aimed at workplace safety and overall worker health [9]. In Sweden and Norway, legislation states that OH services should be available when required by the working conditions. OH service providers are, however, generally not key actors in Swedish sick-listing practice; only a small share of sickness certificates are issued by occupational physicians [10].

In Swedish practice, access to OH services is based on voluntary contracts between employers and OH service providers; approximately 65 % of the working population state that they have access to OH services [11]. It has also been noted that small employers use OH services less than larger employers [5, 12]. Swedish OH services expanded in the 1980s when a financial support system from the state was introduced. At this time, many large companies had internally organized OH service providers. The state support was withdrawn in 1993, and many companies closed their OH service units because of economic recession, which has led to a general decline in access to OH [12]. Attempts have been made to introduce new subsidies for OH providers, but these have not been successfully implemented [13]. Today, the majority (approximately 80 %) of Swedish OH service providers are external companies [12], selling their services on an open market.

### Aim

The aim of this study was to explore how employer representatives and OH professionals describe their business relations and the use of OH services in early rehabilitation and RTW in relation to the organization of such services. The focus of this article is thus on the conditions for involving OH services, rather than on the content of the services.

### Social capital as a theoretical framework

In this study, the literature on social capital is used as a theoretical framework for the analysis. This was chosen based on preliminary analyses indicating that the contractual and interpersonal relationships between employers and OH service providers were important factors in determining the use of services. The literature on social capital was found to have good explanatory value in describing such relationships.

According to a recent definition, social capital may be seen as made up of resources attributed to both individuals and groups (e.g. workplaces or communities), and includes trust, norms, social support, information channels and social credentials [14]. In other definitions, it is emphasized how the norms, values and trust in networks between people or institutions create social structures that facilitate collective and coordinated action [15]. Another distinction is between individual and ecological social capital; the latter is a measure on an aggregated level that may be used for studying social capital within organizations or communities [16]. In this study, social capital is primarily seen as a resource at the group or organizational level, which may benefit individuals as well as groups or organizations.

The concept have been further defined in the literature by distinguishing between structural and cognitive components [17]. In an organizational context, structural social capital refers to networks that give access to resources, which in this study comprises the arrangement for service provision and financing of OH services. The cognitive dimension comprises support, reciprocity, sharing and trust [18]. Some have identified a relational dimension (trust) as separate from the cognitive dimension, whereby the latter is more concerned with shared vision between people and units in an organization, or between organizations [19]. In this study, cognitive and relational social capital may refer to interpersonal relationships and perceptions.

Apart from the structural and cognitive components, three distinct types of social capital have been identified: bonding, bridging and linking [20]. Bonding and bridging social capital refers to horizontal relations of trust and reciprocity between individuals and groups at the same hierarchical level; the former between persons of similar social identities, and the latter between persons from different backgrounds. Linking social capital concerns vertical relationships between people “interacting across explicit, formal or institutionalized power or authority gradients in society” [20:655]. In this study, the relationship between employers and OH providers is primarily concerned with bridging social capital, because it concerns connections between people with different competencies engaging in a mutual endeavour.

Social capital has been proposed to increase efficiency in exchanges and services, whereby the existence of a bond of mutual trust makes it unnecessary to settle on the terms for each exchange [15, 21]. It is further thought to limit opportunism and may build a sense of belonging and shared action [22]. Social capital has been shown to have a strong association with resource exchange and value creation in business settings [23], and to be a key aspect for health, both at work [18, 24, 25] and on a societal level [26]. A study has also noted that social capital may serve a dual function in both promoting health and business performance [27]. It has been suggested that social capital is also important for knowledge transfer, and that the influence of trust for spreading knowledge is greater in inter-organizational than in intra- organizational relationships [28]. Furthermore, inter-organizational partnerships that focus on co-operative processes and long-term supply relations may facilitate the building of social capital, which eventually facilitates the flow of information and reduces transaction costs [22].

Social capital may be measured in different ways. In studies of organizational social capital, it is most commonly measured by an eight-item instrument that makes it possible to differentiate between bonding, bridging and linking social capital [18, 27]. In the business literature, other quantitative measurements have been used to evaluate social networks and trust in relationships, referring to the structural, cognitive and relational components [23]. In qualitative studies, social capital has been used primarily as an explanatory framework, e.g. in case studies or in explorative interview studies.

## Methods

The study is based on 16 individual interviews and 8 focus groups (*n* = 44) with managers with Swedish public employers (municipalities and county council units in the health care sector), and interviews with 25 different professionals from their OH service providers (physicians, physiotherapists, nurses and psychologists). A combination of interviews and focus groups was chosen to achieve a breadth of experiences from several professions and stakeholders.

### Selection of respondents

Participants were chosen purposively to attain a diversity of opinion related to different perspectives of OH provision (i.e. different OH professionals, as well as employers using OH services). OH representatives were identified through a survey study to OH service providers, from which the research group selected providers with customers in municipalities and county councils. Criteria for inclusion were that contracts with providers were in place for at least 6 months ahead, and that these included services related to occupational rehabilitation. The selection was also made to assure variation in terms of geographic location, size and organizational form (internal and external).

Employer representatives were identified through two sources. First, OH service providers identified 16 unit managers or human resources (HR) staff from municipalities or county councils, under the criteria that they had responsibility for rehabilitation and had contacts with the OH provider in rehabilitation cases. These respondents were interviewed individually. Second, data from another study with a similar focus [29] was used to strengthen the employer representation in the data material. In this study, 8 focus groups with 44 municipal managers were held where the municipalities were identified from a statistical database from an insurance company, and the managers were identified by HR staff in each municipality. Inclusion criteria were that they represented municipalities with either high or low sick leave rates. The managers represented different units in the municipalities with a maximum of 50 employees, and all had experience from rehabilitation cases. All municipalities in the focus groups had externally organized OH service providers, and two of the four municipalities had recently outsourced their internal OH service to external providers. Two of the municipalities also purchased HR services from companies specialized in employee assistance programmes, i.e. less medically oriented services than regular OH services, in addition to services purchased from an OH provider.

An overview of the respondents is given in Table 1.

**Table 1:** Overview of respondents

Unit	Organizational form	Customers	Respondents from OH service provider	Respondents from employer
1	External	Municipal	1: Occupational physician	1: Unit manager, elderly care
			2: Organizational consultant	2: Unit manager, day-care centre
2	Internal	County council	1: Occupational physician	1: HR, elderly care administration
3	External	Municipal and county council	1: Occupational physician	1: HR, elderly care administration
			2: Ergonomist	
4	External	Municipal	1: CEO of OH provider	1: HR adviser
			2: Ergonomist	
5	Internal	Municipal	1: CEO of OH provider	1: HR specialist
			2: Rehabilitation coordinator	2: Unit manager, disability care
6	Internal	Municipal	1: CEO of OH provider	1: Unit manager, social services
			2: Occupational nurse	2: Unit manager, day-care centre
				3: Unit manager, elderly care
7	Internal	Municipal	1: Occupational physician	1: HR strategist
			2: Occupational nurse	
8	Internal	Municipal	1: Occupational nurse	1: Unit manager, elderly care
			2: CEO of OH provider	
9	Internal	County council	1: Occupational nurse	1: Rehabilitation coordinator, hospital
			2: CEO of OH provider	
10	Internal	Municipal and county council	1: Occupational physician	1: HR specialist, county council
			2: Psychologist	2: HR consultant, dental care
			3: Occupational nurse	
11	External	Municipal and county council	1: Behavioural scientist	–
			2: Occupational physician	
12	External	County council	1: Occupational nurse	1: HR consultant, dental care
			2: Psychologist	
13	External	Municipality	–	Focus group with unit managers (*n* = 4)
				Focus group with unit managers (*n* = 6)
14	External	Municipality	–	Focus group with unit managers (*n* = 4)
				Focus group with unit managers (*n* = 7)
15	External	Municipality	–	Focus group with unit managers (*n* = 7)
				Focus group with unit managers (*n* = 5)
16	External	Municipality	–	Focus group with unit managers (*n* = 3)
				Focus group with unit managers (*n* = 8)

*CEO* chief executive officer, *HR* human resources

### Data collection

The interviews were semi-structured, focusing on a range of topics regarding the use of OH services in early RTW (e.g. how and by what methods work ability assessments are provided; OH providers’ involvement in work adjustments; cooperation between employers, OH providers and other stakeholders). The interview guide was designed based on the research project’s focus on OH services in early RTW, covering both the actual services and the conditions for service delivery. The focus on work ability assessments and work adjustment were based on findings from the literature on work disability prevention [1, 2]. The interviews were carried out on site at the OH provider or the employer premises. One employer interview was conducted by phone. The interviews lasted for approximately 60 minutes and they were transcribed verbatim.

The focus groups were semi-structured, using an interview guide covering the municipalities’ policies for rehabilitation, what types of RTW interventions they offered to returning workers, and how OH services were used to support RTW processes. Between three and eight persons participated in the focus groups, which were moderated by two researchers and lasted for approximately 60–90 minutes. They were then transcribed verbatim.

Data collection ended when the sample comprised sufficient variety in terms of different professionals within OH, representing internal and external OH services of different sizes.

### Analysis

The coding of the material was done in NVivo (versions 9 and 10), and was both inductive (based on respondents’ answers) and deductive (based on interview questions). Coding was done by two researchers independently and controlled by two more researchers to secure the reliability of the coding. The deductive coding focused on more specific questions from the interview guide, such as what methods for work ability assessments were used. Inductive coding was used for the more open ended parts of the interviews, such as how employers perceived services or what OH professionals would like to focus on in their work.

The material was then analysed using qualitative content analysis [30]. In this process, categories were identified; the prominent ones concerned approaches to and the content of work ability assessments and workplace adjustments; approaches to and routines for sick-listing processes; and the organizational relationships between employers and OH providers. This article focuses specifically on the category of organizational relationships; other parts of the material will be presented in subsequent articles. This delimitation is the result of the analysis process, whereby the questions about organizational conditions were identified as sufficient for a separate study.

Within this subset of the material, themes were identified that comprised how the OH provider was organized (internal or external to the employer), and how this organization was related to the perceived quality of services, including continuity, dialogue and insight into workplace conditions. This part of the analysis was done in an inductive and data-driven way. In the next step, social capital was identified as a central concept to frame the analysis theoretically. The first author’s background in social sciences had a strong influence in the choice of theoretical framework; social capital was chosen because of its explanatory value in terms of business relationships, cooperation and organization of services. Although questions referring to the various dimensions of social capital were not explicitly asked in the interviews, it was possible to identify and categorize responses related to structural, cognitive and relational dimensions of social capital. Illustrative quotes were selected and translated into English.

The trustworthiness of the analysis was secured by constant discussions of emerging results within the group, as well as by consulting a reference group for the project. The reference group was composed by representatives for OH providers, the OH trade organization, public employers and researchers in the field of occupational rehabilitation. Feedback was obtained from the group regarding the design of the project and the results. Preliminary results were also presented at seminars to receive input from professionals in the field. Disagreements were resolved by discussions in the research group.

### Role of the researchers and ethical considerations

None of the researchers are active clinicians in OH services. Two of the authors are social scientists, one a psychologist, one a physician and one an ergonomist. The mixed background of the authors helped the analysis by offering different perspectives.

All participants gave oral informed consent for participation in the study. Written consent was not considered necessary because the study interviewed professionals about aspects of their work. Data were anonymized to maintain confidentiality in the analysis process. The Regional Research Ethics Committee in Stockholm approved of the study (Dnr 2011/141-31/5).

## Results

The results concern how employer representatives and OH professionals experienced their cooperation. The following two broad themes were identified in the material: how OH provision was organized and its relation to service delivery; and the relation between organization and the perceived quality of services, involving continuity in relationships, dialogue about service design and providers’ insight into conditions at the workplace.

### The organization of OH provision

This theme was concerned with how the provision of OH services was organized and its relation to the design and use of services.

#### Internal provision and centralized budgets facilitate the use of OH

A central aspect for the character of contracts and cooperation between employers and OH service providers was whether the OH provider was organized as an internal department within the employer’s organization or if the provider was an external company. Employers with internally organized OH provision generally described a closer and more developed cooperation with the OH provider; the internal organization facilitated easy access to services. Another key factor for the quality of the cooperation was how the contract was regulated. In cases where the costs for OH services were centralized in the employer organization, unit managers did not need to consider the costs for specific interventions, which was perceived as promoting service use. Hence, a structural dimension of the relationship had consequences for how managers perceived the accessibility of OH services; it was seen to be more or less freely available, which was considered as positive:

The OH services are built into the organization, so it’s just to call them, both for employees and as a manager. There’s no limit to that.

*It’s not connected to any charges or anything like that?*

No, and that’s the beauty of it. It’s just to call them. (Employer, HR department)

It may be noted how the use of internal OH services most likely were not without limits in these organizations; the point is how unit managers perceived the accessibility. An internally organized provision with a centralized budget was believed to facilitate the use of OH by reducing hassle and negotiation. One manager specifically noted how an internal OH provider removed financial motives from the decision on whether they would consult OH professionals or not. It is, the manager said, “good not to be concerned with money” (employer, unit manager). However, it may be noted that a centralized budget is not necessarily connected to having an internal OH service provider (and vice versa), although this was the case in this study.

#### Contracts influence the design of services

One of the employers described that they had contracts with both an internal and an external OH provider, and the cost for each specific service or intervention was much clearer in the relationship with the external provider. They had a set price list with limited possibilities for designing services tailored to the needs of the employer, and the invoice for services was delivered directly to the unit, which made the cost more visible. To prevent managers from refraining from purchasing OH services, some employers with external providers financed a basic service provision centrally. In a focus group with municipal managers, however, they described how the municipalities had moved from centrally financed services to placing the costs directly in the unit managers’ budgets, where they expressed concerns that this would prevent managers from using OH services.

A manager from a municipality that had recently outsourced their OH services described how it was difficult to decide whether the services provided by the OH provider were reasonably priced and whether they gave sufficient value for money.

It constantly feels like they are trying to sell me something. A work assessment concept for 15 000 [SEK] as soon as you show them a rehabilitation case, it feels like too much business to me. (Focus group with municipal managers)

OH professionals had different views on service delivery, depending on whether they were internal or external to the employer organization. Although some employers experienced a set list of services as limiting, an OH physician from an external provider expressed how it provided clarity and transparency in their business relations when services were set and priced and employers were limited to specific options.

There was one example in the data of a (rural) municipal employer that described a close relationship with an external OH provider. In this case, the OH provider offered a specified set of services combined with services developed in dialogue with the employer. The rural setting implied that there were few options of providers in the area, leading to close personal contacts that influenced the business relationship. As such, the case was an exception from the general trend in the material, where employers with external OH providers described a more distanced relationship to their provider with less flexibility in services.

#### Competition between OH providers and HR departments

An external OH professional noted how the employers’ HR departments at times could be considered as rivals when HR services overlapped with their work within the employer organization.

Today, I think that the HR department can be a threat or hindrance, since they want to take care of many things. If there’s an HR department, they like to do these things, maybe to save some money by not consulting OH services. (OH, organizational consultant)

This quote also reflects how employers who have outsourced OH services may replace some of the services previously purchased through OH providers with other internal structures, such as HR departments, specifically with regard to services that require less medical expertise. Consulting an OH provider is thus not the only possible option for employers to support their employees in health-related issues. Some employers noted how they had reduced their need for OH services by building such internal structures, whereby OH services were only purchased when there was a need for medical expertise. Some employers with external OH providers also consulted other companies for less advanced health-related services (or employee assistance programmes) that the employees could use without authorization from their managers. In some cases, these companies were considered a cheaper alternative to OH providers.

### Relation between organization of OH and perceived quality in relationships

This theme is concerned with how the organization of OH provision affects the quality of the relationship between employers and OH professionals.

#### Long-term contracts facilitate continuity and familiarity with the workplace

Both employers and OH providers preferred having long-term contracts, because they considered provision of OH services to be facilitated if the provider had good knowledge of the work conditions and environment in the workplace. Employers with external OH provision described how such continuity was difficult to attain when contracts were re-negotiated on an annual or biannual basis. In one case, an employer had decided to keep an internal OH provider for certain units while outsourcing others; in the units kept internal, relations were particularly good and valued, and continuity played a central role in the decision.

Of course we wanted to continue with the county council’s internal occupational health provider. In the latest procurement, it was decided that we should have two companies for occupational health services. It was a political discussion about this, where they decided to keep the internal provider in the social welfare unit, since they had been there for many years. (Employer, HR)

An external OH provider noted how the OH sector was developing towards more specific services and less strategic and continuous cooperation with employers, with the risk of obliterating a joint process in designing services. Geographic proximity was also mentioned as a central factor for developing a close cooperation, and employers appreciated easy access and flexible ways for contacting OH professionals.

In close relationships (predominantly among employers with internal OH providers), the dialogue focused more on the needs at the workplace than on what was included or not in the contract; the mutual agreement was taken for granted and was not re-negotiated. An occupational physician noted how the contract between the parties had little influence over the services offered:

Honestly, I’m not sure I could say whether there is a contract or what it looks like. I know that the private companies all have different contracts, where they always need to check what they are allowed to do. But that’s not the case here. (OH, occupational physician)

#### Involving OH professionals in preventive work requires trust

In some cases, the OH provider was considered a central actor in the employers’ health and safety strategies, whereby they jointly decided on which services were relevant for the employer. They could also assist in advising employers when deciding on purchases of materials or designing new workplaces in order to prevent future occupational hazards. Several OH professionals expressed how they desired to establish such types of close cooperation in preventive work, where they strove to be a strategic partner in health and safety work.

That’s an advantage with being internal, that I participate in the management meetings where we discuss work environmental issues and rehabilitation processes. (OH, CEO)

Both internal and external providers expressed how lacking legitimacy and mandate on a strategic level could complicate such cooperation. Especially the external OH providers described having little influence over such strategic issues. An employer noted how mutual trust and positive relations was key for developing a close cooperation:

We could be much faster in referring sick-listed employees to occupational health services. It’s all about managers knowing how to consult them and when. We could be much better at using their services. […] It’s about information, but also about relationships, that you have to gain that trust. Our provider is well on the way at getting in much earlier. (Employer, HR)

Several employers describe a positive development where their cooperation with their OH provider has helped them in reducing sick leave and utilizing services better and earlier in the sick leave process. This was not related to whether the OH provider was internally or externally organized; one employer noted how the use of an external provider had helped them clarify what the OH provider should help the employer with: they should focus on medical issues and to stay out of issues related to workers’ employment status.

They [the OH provider] get better and better. As an employer, we don’t know so much about medicine, so they do their work on that and do not interfere with employment issues, which they used to do a lot more. Nowadays, their role is to support us in work adjustments and what we need to do. Roles are much clearer now. (Employer, HR)

This employer illustrates how, with some employers, internal HR departments had been able to replace the OH provider in dealing with non-medical issues, and where the OH provider was expected to deliver more medically oriented services. Hence, employers had different attitudes to involving OH providers in close cooperation, where some were interested in using OH as a strategic partner, while others had other structures to deal with prevention.

The access and utilization of OH services depends on the contracts and organization between the employer and the provider, and the results illustrate how employers have different needs and preferences regarding how to manage this – either through internal OH provision where services have a broad scope and are designed in dialogue, or through a separation between internal HR departments and external OH providers specialized in medical services. Independent of this, trustful relationships, continuity, geographic proximity, insight into conditions at the workplace, and clarity about roles were considered important.

## Discussion

The results show how a developed and mutually beneficial relationship between employers and OH providers were related to certain conditions that had structural, relational as well as cognitive aspects. These results are here related to the literature on social capital.

### Social capital facilitates purposeful cooperation between employers and OH providers

In this study, the structural dimension (e.g. the arrangement for service provision and financing) and the relational dimension (e.g. the trustful and flexible communication) of social capital both facilitated and reinforced a purposeful cooperation. The employers’ descriptions of how their access to and use of OH services were facilitated by internally organized provision and centralized contracts illustrate how social capital serves to increase resource exchange (OH services were used often) and facilitate business relations (little concern over contracts and money) [23, 31]. It was more common for employers with external providers to describe suspicion towards the OH provider, especially with regard to value for money where the price for services was a recurring concern.

Previous studies indicate that social capital is important for knowledge transfer, especially in inter-organizational relationships [28]. It was clear in this study that long-term supply relations facilitated the building of social capital, and that this was more easily achieved when OH services were organized internally. There was one case where an external OH provider had developed a similarly close relationship with a municipal employer; however, this may have been related to geography, as it was in a rural area where the number of OH providers was limited and the relationship therefore shared many of the characteristics described by employers with internal OH provision. These results illustrate how social capital may not in itself be the determinant for close relationships. In this case, the interdependency and the social capital were to a large extent a consequence of the contextual conditions that facilitated a closer relationship. This indicates that there is a mutual reciprocity between cooperation and social capital, and these serve to reinforce one another. It is also interesting to note how such closer relationships tended to facilitate service designs based on the needs of the workplace rather than what is in the contract. Quality of services is thus dependent on structural (contracts and organizational form) as well as relational (trust and dialogue) and cognitive (shared vision) social capital. If employers wish to use OH strategically as a partner in rehabilitation and prevention of sick leave, attention to these dimensions is important.

### Relationship between social capital, roles and visions

There are examples in the study of how the social capital, on a structural, relational and cognitive level, influenced how employers and OH providers perceived their roles.

Employers with internal OH providers described how OH professionals took active part in strategic work in the employer organization, where a close and trustful relationship facilitated OH providers being seen as legitimate actors with a mandate to influence strategic decisions. This structurally determined position had an impact also on the cognitive social capital, i.e. the OH providers’ perception of their role and their vision of what they would like to achieve in cooperation with employers.

Employers with external OH providers described much less developed cooperation, with a less developed common vision of the relationship and the work at hand [28]. In these relationships, employers to a larger extent seemed to arrange services traditionally provided by OH through HR or other sources. This also seemed to affect the roles and visions, whereby external OH providers were seen as merely selling services on a market, rather than being strategic partners to the employers. The lower degree of social capital in external relationships (described in terms of suspicion and lack of reciprocity) complicated cooperation, and services were typically limited to predetermined lists of services with fixed prices. In such cases, both the ability and willingness to cooperate was diminished [32].

### Trend towards external OH provision

Although both employers and OH providers appreciated relationships characterized by a high level of social capital, many respondents described how the trend in OH provision was precisely the opposite. In recent decades, increasing numbers of both public and private employers have chosen to outsource OH provision [12], and our results suggest that some of the services previously carried out by OH departments are now sometimes managed by HR departments. Some employers described how outsourcing clarified the role of OH providers as medical expertise, whereas others complained about the lack of flexibility in services such outsourcing could imply.

This increasing marketization of OH services implies that OH providers need to compete either through the type of services they are offering or through prices. Problems may arise in the supply as well as in the demand of services, where the relevance of the services offered is dependent on the employers’ abilities to express their needs, as well as OH providers’ abilities to adjust their services to specific conditions in the workplace. Although this could be expected to imply more flexibility in services, our results indicate the opposite: that OH providers resort to offering predetermined services that are not developed in dialogue with the employer. This may be further complicated by short-term contracts between the parties, where there is not enough time to develop a sufficiently close relationship. Another aspect of short-term contracts, where social capital is lower, may be less opportunities and time for evaluating services, and thus reduced possibilities for quality improvements [28].

Occupational health and safety is one of the most common HR functions that companies choose to outsource [33]. The “make or buy” decision (to organize and provide internal services or to buy from external providers) is often influenced by companies striving to reduce costs and to focus on core activities, or by ambitions to increase quality by contracting expert services while focusing internal HR systems on more strategic tasks [34, 35]. This was an obvious feature in our study; some employers described the OH services as primarily an expert function, while other services were carried out by the HR department, where costs was an issue. The importance of transaction costs in outsourcing decisions has been noted in other studies [36], but it has also been pointed out that using internal OH services may be cost-efficient in the longer term with quality improvements and reduced workers’ compensation claims by incorporating previously external clinical services into an already existing internal OH service department [37]. A recent study also pointed out that the sum employers spend on rehabilitation is often small compared with the costs of production loss when employees are sick-listed [38], which indicates that there may be financial incentives to improve strategies for rehabilitation and RTW. In our study, the results suggest that a relationship characterized by a high degree of social capital means less suspicion and more flexibility in services, which according to previous research improves efficiency and collective action [23]. This may, in turn, imply lower long-term costs.

It may also be asked what the possible consequences are of relying on internal HR departments in matters where medical knowledge may be called for. Although employers described how they consulted OH professionals when needed, it is likely that managers will hesitate to do so unless it is perfectly clear that the problem needs medical expertise, with the consequence that OH services are consulted less often. Thus, a consequence would be that medical knowledge is given a less prominent role in dealings with employees’ health problems.

The increasing reliance on market-based solutions for provision of OH services has had consequences for how OH services are organized and designed, and this study indicates decreasing possibilities for tailoring interventions to specific workplace needs. Employers with external OH providers could possibly benefit from increasing the integration between OH services and the workplace environment through establishing longer contracts promoting dialogue regarding services. Furthermore, centralizing costs for OH services within the employer organization seems to promote use of OH services, which may also be a possibility for employers with external OH providers. These are questions that need to be considered in decisions on how to organize OH provision, where the added value of OH services for employers and how this is influenced by contractual arrangements need to be clearly identified.

### Methodological considerations

The study had a qualitative and explorative character, and the results should be considered in light of the sample. The employers were all public (municipalities and county councils), which limits the transferability to other sectors. The legislative framework for OH provision differs greatly between countries, which implies that the results are primarily transferable to contexts in which OH provision is offered voluntarily in a market-based system. In the material, there was a surplus of internally organized OH service providers relative to the distribution in the Swedish context, and it is possible that a broader sample of external OH providers would reveal examples of external providers with a close and long-term relationship with employers. There may also have been selection bias, because the OH providers may have identified employer representatives with whom they had better relations. For the focus groups, however, employers were recruited through other sources. Another possible bias in the material is that, while Swedish OH has been heavily outsourced, the internal providers that are left may be the more successful ones. Nevertheless, the theoretical framework of social capital adds to the transferability of the results by pointing to conditions that are of relevance regardless of the organization of OH provision.

A possible limitation of the study is that social capital was not explicitly measured, because the concept was used as an explanatory framework for an inductively derived research question. This implies that all aspects of social capital were not covered; mostly, the study focuses on the relationship between structural social capital (through organization of services) and how employers and OH providers perceived the quality of their relationships (referring to some but not all aspects of cognitive and relational social capital).

## Conclusions

In this study, relationships with a high degree of social capital were considered positive for purposive use of OH services in rehabilitation and RTW. Such relationships had structural, relational and cognitive elements. The structural dimension comprised continuity in contracting, geographic proximity and providers’ insight into conditions in the workplace. The relational dimension concerned trust, reciprocity, flexibility in service delivery and an extensive dialogue between employers and OH professionals. The cognitive dimension concerned a shared vision of the cooperation between employers and OH service providers, i.e. whether OH providers were perceived as engaged in supporting the employer or primarily interested in fulfilling their own goals (e.g., selling services). The results illustrate how the structural social capital interacts with and reinforces cognitive and relational social capital by affecting the perceived roles and visions of employers and OH providers.

In this study, favourable conditions for social capital were more easily met in cases where OH services were internally organized. Relationships between employers and external OH providers were described as less developed, whereby the employers expressed more suspicion towards their providers. As outsourcing of OH services is a common trend in the Swedish system, attention to social capital is called for when contracting such services.

Future studies may focus more explicitly on the relationship between social capital and professionals’ perceptions of their roles, or how the organization of services affects the conditions for well-functioning OH provision.
